# Spatial and temporal changes in the PGE2 EP2 receptor in mice hippocampi during postnatal development and its relationship with cyclooxygenase-2

**DOI:** 10.22038/ijbms.2021.56286.12556

**Published:** 2021-07

**Authors:** Hyo Young Jung, Woosuk Kim, Kyu Ri Hahn, Sung Min Nam, Sun Shin Yi, Hyun Jung Kwon, Min Soo Kang, Jung Hoon Choi, Dae Won Kim, Yeo Sung Yoon, In Koo Hwang

**Affiliations:** 1 Department of Veterinary Medicine & Institute of Veterinary Science, Chungnam National University, Daejeon 34134, South Korea; 2 Department of Anatomy, College of Veterinary Medicine, and Veterinary Science Research Institute, Konkuk University, Seoul 05030, South Korea; 3 Department of Anatomy and Cell Biology, College of Veterinary Medicine, and Research Institute for Veterinary Science, Seoul National University, Seoul 08826, South Korea; 4 Department of Anatomy, School of Medicine, Wonkwang University, Iksan 54538, South Korea; 5 Department of Biomedical Laboratory Science, College of Medical Sciences, Soonchunhyang University, Asan 31538, South Korea; 6 Department of Biochemistry and Molecular Biology, Research Institute of Oral Sciences, College of Dentistry, Gangneung-Wonju National University, Gangneung 25457, South Korea; 7 Department of Anatomy, College of Veterinary Medicine and Institute of Veterinary Science, Kangwon National University, Chuncheon 24341, South Korea

**Keywords:** Dentate gyrus, Growth and development, Hippocampus, Mice, Prostaglandin E EP2 - subtype

## Abstract

**Objective(s):**

Prostaglandin E2 E-prostanoid 2 receptor (PGE2 EP2), downstream of cyclooxygenase-2 (COX-2), plays an important role in inflammatory responses, but there are some reports about synaptic functions of COX-2 and PGE2 EP2 in the hippocampus.

**Materials and Methods:**

C57BL/6J mice were sacrificed at postnatal days (P) 1, 7, 14, 28, and 56 for immunohistochemical staining for EP2 and doublecortin as well as western blot for EP2. In addition, COX-2 knockout and its wild-type mice were euthanized for immunohistochemical staining for EP2.

**Results:**

EP2 immunoreactivity was observed in the majority of the cells in the dentate gyrus at P1 and P7, while at P14, it was detected in the outer granule cell layer and was confined to its subgranular zone at P28 and P56. EP2 protein levels in the hippocampal homogenates were also highest at P7 and lowest at P56. EP2 immunoreactivity was partially colocalized, with doublecortin (DCX)-immunoreactive neuroblasts appearing in the mid-zone of the granule cell layer at P14 and in the subgranular zone of the dentate gyrus at P28. Co-localization of EP2 and DCX was significantly decreased in the dentate gyrus in the P28 group compared with that in the P14 group. In COX-2 knockout mice, EP2 immunoreactivity was significantly decreased in the hippocampal CA1 region (*P*=0.000165) and dentate gyrus (*P*=0.00898).

**Conclusion:**

EP2 decreases with age, which is expressed in DCX-immunoreactive neuroblasts in the dentate gyrus. This suggests that EP2 is closely linked to structural lamination and adult neurogenesis in the dentate gyrus.

## Introduction

Phylogenetically, the hippocampus is the oldest region in the brain, but it is an important region of the limbic system and is associated with functions of learning and memory (1). Being an archicortex, the hippocampus consists of three cortical laminae and demonstrates the capacity for structural reorganization (2). This conforms to the fact that, during embryogenesis, granule cells in the hippocampal dentate gyrus constitute only 15% of the total cell count; however, the cells proliferate and migrate into the granule cells 2 weeks after birth (3, 4). Moreover, the granule cells are generated continuously by the process of adult hippocampal neurogenesis in which cells located in the subgranular zone of the dentate gyrus can form granule cells by the process of proliferation, migration, and integration into the granule cell layer (5, 6). In a previous study, we demonstrated that neuroblasts are found abundantly in the granule cell layer of the dentate gyrus 2 weeks post birth and are seen in the subgranular zone of the dentate gyrus only after 3 weeks of birth (7).

Various factors regulate the morphological lamination of the hippocampus and adult hippocampal neurogenesis in mice during postnatal development. Cyclooxygenases (COXs) produce prostaglandins (PGs) from arachidonic acid and occur in two different isoforms: COX-1 and COX-2 (8). Constitutive levels of COX-1 are found in cells, while COX-2 is strongly expressed in response to pro-inflammatory challenges (8, 9). COX-2 catalyzes the production of PGH_2_ and prostanoids, including PGE_2_, PGI_2_, and thromboxane A_2_, from arachidonic acid in the cell membranes (10). However, several lines of evidence demonstrate that COX-2 is detectable at base levels in the brain (7, 11-14), and administration of COX-2 inhibitor or COX-2 depletion in mice significantly reduces the number of Ki67-positive proliferating cells and doublecortin (DCX)-positive neuroblasts in the dentate gyri of adult mice (15-17). COX-2 inhibition also impairs memory formation and synaptic transmission in rats (18). Conversely, PGE_2_ facilitates glutamate release and excitatory synaptic transmission (19), and administration of sulprostone, an analog of PGE_2_, increases the number of 5-bromo-2’-deoxyuridine-positive dividing cells in the subgranular zone of the dentate gyrus (20). Additionally, endogenous PGE_2_ plays an important role in memory acquisition as well as synaptic plasticity (18). The action of PGE_2_ is mediated by binding to four distinct G-protein coupled E-prostanoid receptors (EP1-4) (21), and the regulation of synaptic plasticity is mediated by the binding of PGE_2_ to EP2 receptor in the cerebral cortex and hippocampus (22).

Although COX-2 and PGE_2_ are closely associated with hippocampal neurogenesis in the brain, little is known about the role of PGE_2_ EP2 receptor expression during postnatal development. Therefore, in this study, we examined the temporal and spatial changes of PGE_2_ EP2 receptors in mice hippocampi and then compared the expression of the PGE_2_ EP2 receptor with doublecortin-immunoreactive neuroblasts to investigate the relationship of EP2 and hippocampal neurogenesis in mice hippocampi. In addition, we also examined the expression of the EP2 receptor in the hippocampi of COX-2 knockout (KO) and its wild-type (WT) mice to elucidate the relationship between COX-2 and EP2 in adult neurogenesis.

## Materials and Methods

***Experimental animals***

Twenty pregnant C57BL/6J mice (15- to 18-week-old) were purchased from Orient Bio Inc. (Seongnam, South Korea) and the pregnant mice were individually housed in cages with paper bedding. The day of birth was considered as postnatal day 0 (P0) and the litters were randomly culled to preserve ten pups per litter as described in the previous studies (7, 14). To investigate the expression of EP2 in the hippocampus, COX-2 KO and its WT mice (8-week-old) were purchased from Taconic (Rensselaer, NY, USA) as described in a previous study (14, 16). The experimental protocol was approved by the Institutional Animal Care and Use Committee (IACUC) of Seoul National University and Kangwon National University.

***Tissue processing***

For postnatal expression of EP2 in the hippocampus, mice at P1, 7, 14, 28, and 56 were euthanized with a mixture of alfaxalone (Alfaxan, 75 mg/kg; Careside, Seongnam, South Korea) and xylazine (10 mg/kg; Bayer Korea, Seoul, South Korea). The brains were quickly removed from the cranial cavity and the brain tissues were fixed with 10% neutral buffered formalin according to previous studies (7, 14). For COX-2 KO and WT mice, the animals at P80 were deeply anesthetized with a mixture of 75 mg/kg alfaxalone and 10 mg/kg xylazine and transcardially perfused with 0.1 M phosphate-buffered saline (PBS, pH 7.4), followed by 4% paraformaldehyde in 0.1 M phosphate buffer (PB, pH 7.4), as described previously (14). Post-fixed brains in the same fixative for 12 hr were embedded with paraffin, as protocols described in the previous studies, and 3-μm coronal sections were mounted onto silane-coated slides with a microtome (Leica, Wetzlar, Germany).

***Immunohistochemistry for EP2***

Immunohistochemical staining was conducted under the same conditions, as described previously (13, 14). Briefly, the sections were hydrated with alcohol series and the endogenous peroxidase removed with reaction to 0.3% H_2_O_2_ in PBS for 30 min. The sections were exposed to heat with citrate buffer (pH 6.0) in a 2100-retriever (Prestige medical, Lancashire, UK) and sequentially incubated with diluted rabbit anti-EP2 (1:1,000; Abcam, Cambridge, UK) antibody for 48 hr at 4 °C. All sections were visualized with chromogen (3,3′-diaminobenzidine tetrachloride, Sigma, St. Louis, MO, USA) in a 0.1 M Tris-HCl buffer (pH 7.2) solution.

***Double immunofluorescence***

To confirm the colocalization of EP2 and neuroblasts during postnatal development, the double immunofluorescence staining was conducted in the hippocampus at P14 and P28. Briefly, the sections were incubated with a mixture of rabbit anti-EP2 (1:200) and goat anti-DCX (diluted 1:25; Santa Cruz Biotechnology, Santa Cruz, CA, USA) overnight at 25 °C. Thereafter, the sections were visualized with fluorescent-conjugated antibodies of FITC-conjugated donkey anti-rabbit IgG (1:600; Jackson ImmunoResearch, West Grove, PA, USA) and Cy3-conjugated donkey anti-goat IgG (1:600; Jackson ImmunoResearch) for 2 hr at 25 °C. Double immunoreactive structures were observed in the dentate gyrus under the confocal microscope using a 20× lens (LSM510 META NLO, Carl Zeiss, Göttingen, Germany).

***Western blot analysis***

Quantification of EP2 protein levels was done within the hippocampus of P1, 7, 14, 28, and 56 mice (*n*=5 per group). Briefly, the animals were euthanized and the denatured hippocampal proteins were loaded onto a polyacrylamide gel, and proteins-transferred nitrocellulose membranes were obtained. The membrane was incubated with a rabbit anti-EP2 antibody (diluted 1:500) and visualized by an enhanced luminol-based chemiluminescent kit (Pierce Chemical). For internal loading control, the membrane was also reprobed with an antibody against β-actin, and data were normalized to the β-actin level in each lane. *Western blotting* was performed in *triplicate*.

***Data analysis***

To quantify the EP2 immunohistochemical data, digital images were captured including granule cell layer and subgranular zone of dentate gyrus with a BX51 light microscope (Olympus, Tokyo, Japan) equipped with a digital camera (DP72, Olympus). Captured images were calibrated into 0-255 grayscale and the grayscale, as well as pixel number, were measured using the ImageJ v. 1.80 software (National Institutes of Health, Bethesda, MD, USA), as described previously (13, 14). Optical density (OD) was calculated using the equation: OD = log (256/mean gray level) and ODs from all sections (*n* = 10) of all of the mice (*n* = 5) were averaged. Data was normalized to the ratio of relative OD (ROD) vs P1 or WT mice.

To quantify the colocalization of EP2 and DCX, Manders’ coefficient was calculated by ImageJ which detects the portion of signals above background threshold from both channels from all sections (*n* = 10) of all mice (*n* = 5).

***Statistical analysis***

The quantified EP2 immunoreactivity and protein levels were statistically analyzed by one-way analysis of variances followed by either Bonferroni’s *post-hoc* test or the unpaired *t*-test using the GraphPad Prism 5.01 software (GraphPad Software, Inc., La Jolla, CA, USA). 

## Results

***Changes in EP2 immunoreactivity during postnatal development***

**Figure 1:**
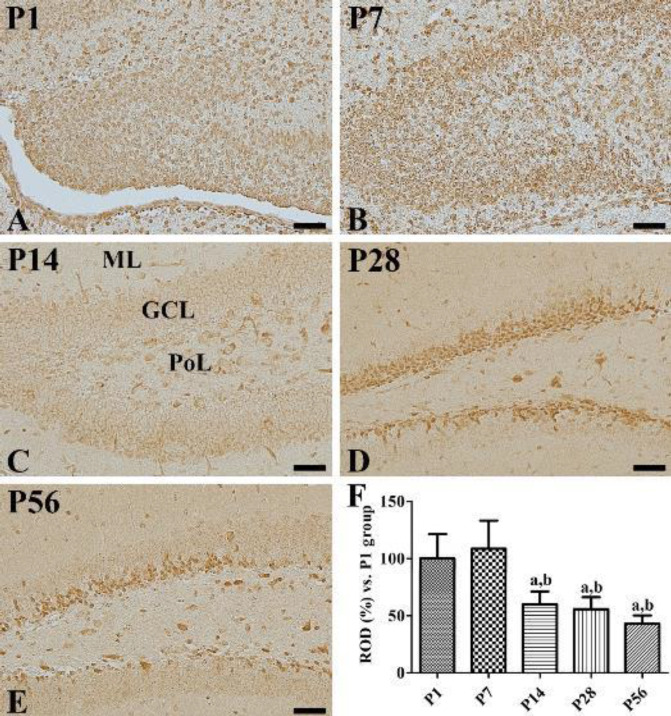
Immunohistochemical staining of PGE2 EP2 in the dentate gyrus at P1 (A), P7 (B), P14 (C), P28 (D), and P56 (E). EP2 immunoreactivity is found in many cells in the dentate gyrus at P1. Note that EP2 immunoreactive structures are found in the granule cell layer (GCL) of the dentate gyrus by P14 and subgranular zone of the dentate gyrus at P28 and P56. ML, molecular layer; PoL, polymorphic layer. Scale bar = 50 μm. (F): ROD is expressed as a percentage of the EP2 immunoreactivity detected at P1 in the dentate gyrus for each section (n=5 per group; a*P*<0.05, significantly different from P1; b*P*<0.05, significantly different from P7). All data are represented as the mean ± standard deviation

In the P1 group, EP2 immunoreactive cells were abundantly observed in the dentate gyri (Figure 1A), while in the P7 group, many EP2 immunoreactive cells were found in the granule cell layers and polymorphic layers of the dentate gyri (Figure 1B). Compared with the P1 group, this group exhibited a slight, though not significant, increase in the EP2 immunoreactivity (Figure 1F). In the P14 group, EP2 immunoreactive cells were observed in the outer granule cell layers and polymorphic layers of the dentate gyri (Figure 1C), and a significant decrease in the EP2 immunoreactivity was observed in comparison with the P1 or P7 groups (Figure 1F). In P28 and P56 groups, EP2 immunoreactivity was observed in the subgranular zones and polymorphic layers of the dentate gyri (Figures 1D and 1E). EP2 immunoreactive cells were particularly found in high abundance in the suprablade regions of the dentate gyri as compared with those in their infrablade regions. EP2 immunoreactivity was similar in the P28 group compared with that in the P14 group, while it was significantly decreased in the P56 group when compared with the P28 group (Figure 1F).

***Localization of EP2 immunoreactive cells in neuroblasts***

**Figure 2:**
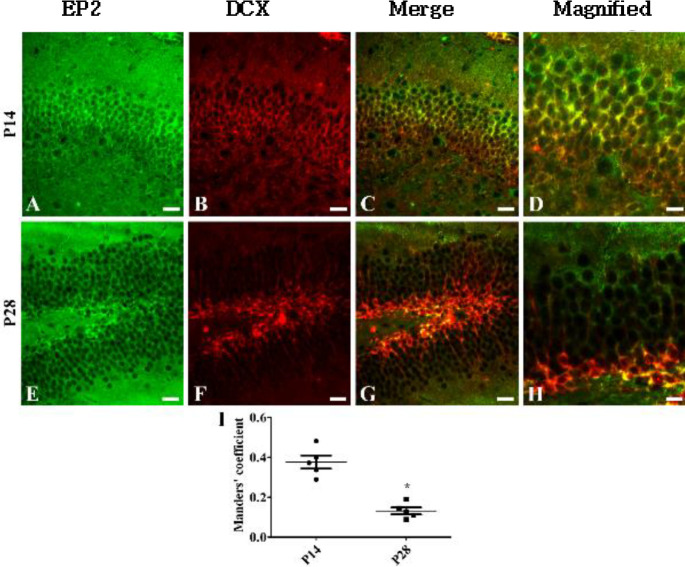
Immunofluorescent staining of PGE2 EP2 (green, A, and E) and DCX (red, B, and F) in the dentate gyrus at postnatal day 14 (P14, A-D) and P28 (E-H). Merge images (C, D, G, and H) are also shown. Note that EP2 and DCX double-labeled cells are found in the mid-zone of the granule cell layer (GCL) of the dentate gyrus at P14, while EP2 and DCX double-labeled cells at P28 are detected in the subgranular zone of the dentate gyrus. Scale bar = 50 μm. (I) Manders’ coefficient analysis of colocalization of EP2 and DCX in the dentate gyrus (n=5 per group; *P<0.05, significantly different from P14). All data are represented as the mean ± standard deviation

To elucidate the localization of EP2 in DCX-immunoreactive neuroblasts, a double immunofluorescent study for EP2 and DCX was performed for P14 and P28 groups. In the P14 group, EP2 immunoreactivity was mainly observed in the outer granule cell layers and polymorphic layers of the dentate gyri (Figure 2A), while DCX immunoreactivity was observed in their inner granule cell layers (Figure 2B). EP2 and DCX double-labeled cells were observed in the central regions of the granule cell layers (Figures 2C and 2D). The subgranular zones of the dentate gyri in the P28 group mainly exhibited EP2 and DCX immunoreactivity as well as an abundance of EP2 (Figure 2E) and DCX double-labeled cells (Figures 2F-2H). In the P28 group, the portion of EP2 and DCX colocalization was significantly decreased in the dentate gyrus compared with that in the P14 group (Figure 2I).

***Changes in EP2 protein levels during postnatal development***

**Figure 3:**
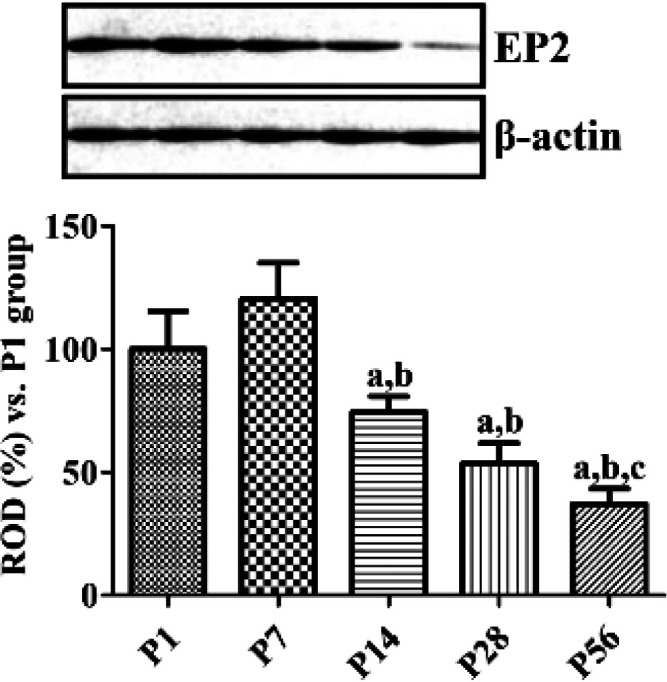
Western blot analysis expressed as a percentage of the value of the prostaglandin PGE2 EP2 immunoblot band at P1. Data were normalized to the β-actin level in each lane (n=5 per group; a*P*<0.05, significantly different from P1; b*P*<0.05, significantly different from P7; c*P*<0.05, significantly different from P14). All data are represented as the mean ± standard deviation

Similar levels of EP2 protein were observed in the hippocampal homogenates of P1 and P7 groups, although they were highest in the P7 group. Thereafter, EP2 protein levels decreased subsequently with age and were lowest in the P56 group. It was observed that EP2 protein level in the P56 group was 37.0% of the level found in the P1 group (Figure 3). 

***Changes in EP2 immunoreactivity in COX-2 KO and WT mice***

**Figure 4:**
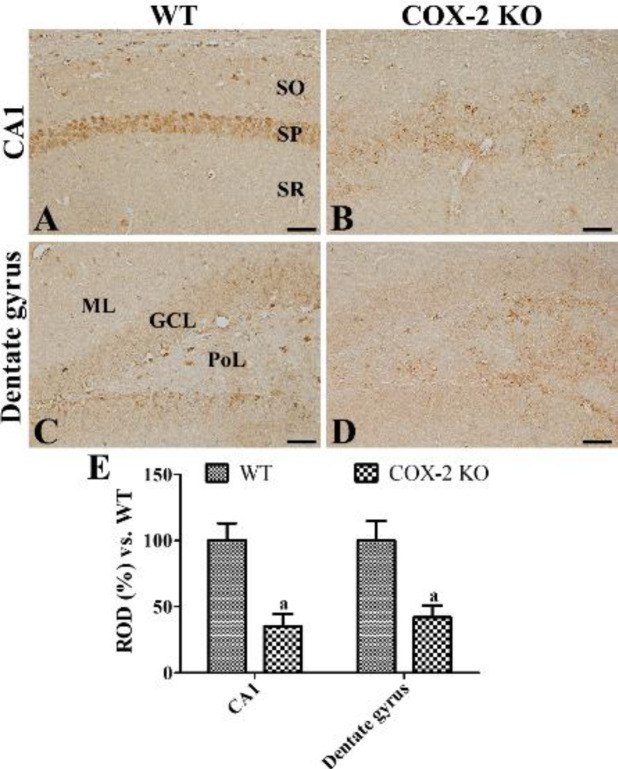
Immunohistochemical staining for PGE2 EP2 in the hippocampal CA1 region (A and B) and dentate gyrus (C and D) of WT (A and C) and COX-2 KO (B and D) mice at postnatal day 80. Note that EP2 immunoreactivity is mainly observed in the stratum pyramidale (SP) of hippocampal CA1 region as well as subgranular zone and granule cell layer (GCL) of the dentate gyrus in WT mice, while in COX-2 KO mice EP2 immunoreactivity is significantly decreased in the hippocampal CA1 region and dentate gyrus. ML, molecular layer; PoL, polymorphic layer; SO, stratum oriens; SR, stratum radiatum. Scale bar = 50 μm. (E): ROD is expressed as a percentage of the EP2 immunoreactivity detected at WT in the hippocampal CA1 region and dentate gyrus for each section (n=5 per group; a*P*<0.05, significantly different from WT mice). All data are represented as the mean ± standard deviation

In the COX-2 WT mice, EP2 immunoreactive cells were observed in the dentate gyri and CA1 region of hippocampi (Figures 4A and 4C). Especially, EP2 immunoreactivity was found in the non-pyramidal cells of the CA1 region and subgranular zone and polymorphic layer of the dentate gyrus. In the COX-2 KO mice, EP2 immunoreactive structures were found in the stratum pyramidale of the CA1 region and polymorphic layer of the dentate gyrus (Figures 4B and 4D). In this group, EP2 immunoreactivity was significantly decreased in the hippocampal CA1 region and dentate gyrus compared with those in the WT mice, respectively (Figure 4E).

## Discussion

In the previous studies, we have demonstrated that COX-2, expressed constitutively in the hippocampus, is associated with hippocampal neurogenesis and synaptic plasticity in the dentate gyrus (14). In the current study, we extended our observations to the PGE_2_ EP2 receptor, which is downstream of COX-2, in the hippocampus. EP2 immunoreactivity was observed in the majority of the cells of the dentate gyri in the P1 group and granule cells of dentate gyri in the P7 group. EP2 immunoreactivity was diminished age-dependently in the dentate gyri by P56 and was limited to their subgranular zones and polymorphic layers (Figures 1 and 3). This distribution pattern of EP2 in the dentate gyri was similar to that of the DCX-immunoreactive neuroblasts as EP2 and DCX immunoreactivities were observed in abundance in the cells located in the dentate gyri at P1 and P7 and were mainly observed in their subgranular zones at P28 and P56. In the present study, we observed that the EP2 expression decreased during lamination of the dentate gyri at P14. Additionally, EP2 immunoreactivity was observed in the outer granule cell layers of the dentate gyri, while DCX immunoreactive neuroblasts were found in their inner granule cell layers (Figure 2). These results are supported by previous studies in which COX-2 immunoreactivity was found in the outer granule cell layers of the dentate gyri at P7 and P14 (14), and DCX immunoreactivity was observed in their inner granule cell layers (7). This result suggests that a decrease in EP2 expression may be associated with completion of structural lamination in granule cells. At P28 or P56, EP2 is mainly expressed in the neuroblasts present in the subgranular zones of the dentate gyri after full development or lamination of dentate gyri. This result is partly consistent with a previous study that determined that EP2 immunoreactivity was co-expressed in the neural progenitor cells, DCX-immunoreactive neuroblasts, and mature neurons (23).

In the present study, we observed less EP2 receptor immunoreactivity in the hippocampal dentate gyrus in the COX-2 WT mice at P80 compared with that in the P56 group although we did not analyze EP2 immunoreactivity quantitatively in this study (Figure 4). This result reflects that EP2 immunoreactivity is decreased with aging, which is consistent with reduction of adult neurogenesis in the hippocampal dentate gyrus (24-27). We also observed significantly decreased expression of EP2 receptor immunoreactivity in the CA1 region and dentate gyrus in the COX-2 KO mice compared with that in the COX-2 WT mice (Figure 4). This result suggests that reduction of EP2 in the subgranular zone of dentate gyrus may be associated with decreases in the neurogenesis of COX-2 KO mice because depletion of COX-2 significantly decreases the proliferating cells, differentiating neuroblasts, and synaptic plasticity (14, 16). PGE_2_ increases synaptic activity in the hippocampal neurons by activation of EP2-mediated signaling (19). Electrophysiological studies demonstrated that PGE_2_ and its EP2 receptor are involved in synaptic transmission and long-term plasticity in the hippocampus (19, 28-31). Exogenous application of PGE_2_ but not of PGD_2_ or PGF_2α_ ameliorates reduction of postsynaptic membrane excitability and long-term potentiation in the granule cells of hippocampal dentate gyrus induced by COX-2 inhibitor (28). In addition, several studies demonstrated the positive feedback loop in COX-2 and EP2 expression in various disease models (32, 33). PGE_2_ increases cell proliferation in the dentate gyrus, growth in embryonic stem cells (20), neurite outgrowth in sensory neurons (34), and sensory neuron-like ND7/23 cells (35). Additionally, treatment with the PGE_2_ EP2 receptor agonist increases the number of neurites in neurons of control mice and mice suffering from Huntington’s disease (36). Depletion of EP2 causes memory impairments in various paradigms of behavioral studies and electrophysiology at the hippocampal perforant path (30, 31).

In this study, we could not elucidate the possible mechanisms of EP2 that could be involved in the structural lamination of granule cells and adult hippocampal neurogenesis. One of the possible mechanisms of EP2 is the activation of cAMP/ protein kinase A (PKA)/ cAMP response element-binding protein (CREB) signaling because EP2 receptor, when coupled with Gαs protein, stimulates adenylate cyclase activity (21), which activates the cAMP/PKA/CREB pathway (37). Administration of PGE_2_ EP2 receptor agonist induces phosphorylation of CREB in the mouse hippocampus 1 hr post-treatment (36). Additionally, we have shown that phosphorylated CREB was expressed in the majority of the granule cells at P7 and was found only in the subgranular zones of the dentate gyri at P21 (38). Moreover, pCREB expression was largely co-labeled with DCX-immunoreactive neuroblasts in the subgranular zones of the dentate gyri (38).

## Conclusion

PGE_2_ EP2 receptor is constitutively expressed in the granule cells of the dentate gyrus and decreases with age by P14 in the dentate gyrus and is mainly expressed, thereafter, in the immature neuroblasts expressing DCX. These results suggest that EP2 expression may be associated with adult hippocampal neurogenesis in the mice during postnatal development.
